# The hallmark and crosstalk of immune cells after intracerebral hemorrhage: Immunotherapy perspectives

**DOI:** 10.3389/fnins.2022.1117999

**Published:** 2023-01-12

**Authors:** Wenqing Zhang, Qingyuan Wu, Shilei Hao, Shengli Chen

**Affiliations:** ^1^School of Medicine, Chongqing University, Chongqing, China; ^2^Department of Neurology, Chongqing University Three Gorges Hospital, Chongqing, China; ^3^Key Laboratory of Biorheological Science and Technology, Ministry of Education, College of Bioengineering, Chongqing University, Chongqing, China

**Keywords:** immune cells, hallmark, crosstalk, neuroinflammation, intracerebral hemorrhage

## Abstract

Intracerebral hemorrhage (ICH) is one of the most dangerous types of strokes with a high morbidity and mortality rate. Currently, the treatment of ICH is not well developed, mainly because its mechanisms are still unclear. Inflammation is one of the main types of secondary injury after ICH and catalyzes the adverse consequences of ICH. A large number of immune cells are involved in neuroinflammation, such as microglia, astrocytes, oligodendrocytes, lymphocytes, macrophages, and neutrophils. Nevertheless, the characteristics and crosstalk of immune cells have not been fully elucidated. In this review, we endeavor to delve into the respective characteristics of immune cells and their interactions in neuroimmune inflammation, and further elucidate favorable immunotherapeutic approaches regarding ICH, and finally present an outlook.

## 1. Introduction

Spontaneous intracerebral hemorrhage accounts for 15–20% of strokes, with high morbidity, incapability, and low survival rates. This tendency is burgeoning year by year and getting younger (Ren et al., 2020; Collaborators, 2021). Currently, clinical treatment for cerebral hemorrhage can be surgical mechanical embolization or minimally invasive removal for sizable hematomas (Singh et al., 2020), and medical treatment is based on blood pressure reduction and hemostatic therapy (Rosand, 2021). Nonetheless, hematoma after ICH can catalyze primary and secondary injury, which could result in unsatisfactory treatment and prognosis for patients (Al-Kawaz et al., 2020). Primary injury is usually generated by the hematoma after cerebral hemorrhage (Green et al., 2021), and the severity of the injury is linked with the size, location, and extent of the hematoma (Zheng et al., 2022). In contrast, the elements affecting secondary injury are sophisticated (Zhu et al., 2019), which encompass edema (Cannarsa et al., 2022), cytotoxicity, excitotoxicity (Zhao et al., 2021), oxidative stress (Song et al., 2021), neuroinflammation (Gu et al., 2021), and so on.

The complex immune and inflammatory cascade response after brain hemorrhage is an adverse aspect causing secondary injury (Loan et al., 2022). During the onset of neuroinflammation, several cytokines and chemokines conduce to activation of microglia and astrocytes and infiltration of leukocytes, and these immune cells participated in the inflammatory response simultaneously secrete inflammatory factors to foster or inhibit the development of inflammation (Zhu et al., 2019). The study of the characteristics, functions, and interactions of immune cells after cerebral hemorrhage has become one of the hot research topics in the neurological field, and more and more studies are attempting to find effective targets for the treatment of cerebral hemorrhage diseases by studying their mechanisms and thus finding or designing relevant drugs to treat intracerebral hemorrhage (Zille et al., 2022). Immune cells contained in the inflammatory response after cerebral hemorrhage and their interactions are the focus of this review, and we also encapsulated the clinical therapy of intracerebral hemorrhage, especially for neuroinflammatory, which can better reflect the process of cerebral hemorrhage from pathophysiological mechanism to clinical transformation and provide a valuable reference for subsequent studies (Tschoe et al., 2020).

## 2. Immune cells in neuroinflammation

The immune cells embraced in brain hemorrhage are ordinarily divided into two categories: One is neuroglia such as microglia, astrocytes, and oligodendrocytes, which are inherent in the brain tissue and involved in the innate immune response. The other is leukocytes such as lymphocytes, monocytes-macrophages, and neutrophils, which are recruited from the blood circulation to the site of brain hemorrhage and play an impressive role in acquired immune reaction (Zou et al., 2021). Based on previous studies, we have recapitulated the hallmark and functions of these cell types, please refer to Table 1.

**TABLE 1:** Markers and functions of immune cells.

Immune cells	Phenotype	Markers	Functions
Microglia/macrophages	M1	CD16, CD32, CD68, CD86, MHCII IL-1β, IL-2, IL-6, IL-12, IL-23, TNF-α, IFN-γ, ROS CCL3, CCL4, CCL5, CCL12, CCL20 CXCL1, CXCL2, CXCL4, CXCL10 CCR1, CCR5, CXCR4 STAT1, STAT3 MMP3, MMP9, MMP12 TLR2, TLR4, TREM-1, HMGB1	Pro-inflammatory Recruit peripheral immune cells Neuronal apoptosis Blood-brain barrier disruption
	M2a	CD36, CD206 IL-4, IL-13, TGF-β Ym-1, Arg-1, FIZZ1, PPARγ Nrf2, TREM-2, STAT6 rCCL17, CCR4 VEGF, PDGF, BDNF	Anti-inflammatory Enhance phagocytosis of hematoma
	M2b	IL-10, CD86, MHCII	Pro-inflammatory Anti-inflammatory
	M2c	IL-10, CD163 Ym-1, Arg-1, FIZZ1	Synaptic remodeling Matrix deposition
Astrocytes	A1	IL-1β, IL-15, TNFα, PAR1	Neuronal death Perihematomal edema
	A2	IL-6, CD147, TGFβ Mt, HN, Cx43, AQP4	Hematoma clearance Tissue repair
Oligodendrocytes		Olig1/2, NKx2.2, Sox10 TLR4, Tmem10	Promote differentiation Iron clearance
Lymphocytes	Th1	T-bet, IFN-γ, IL-2	Cellular immunity
	Th2	IL-4, IL-5, IL-9, IL-13	Macrophage polarization toward M2
	Th17	IL-17, IL-21, IL-22, IL-23 TGF-β, RORγt	Immune regulation Pro-inflammatory
	Treg	CD25, CD127, Foxp3	Anti-inflammatory
Neutrophils		TNF-α, ROS, MMP-9 NETs	Exacerbate brain injury

Abbreviation: IL, interleukin; TNF-α, tumor necrosis factor-α; IFN-γ, interferon-γ; MMP, matrix metalloproteinase; TGF-β, transforming growth factor-β; MHCII, major histocompatibility complex II; ROS, reactive oxygen species; TLR, toll-like receptor; CCL, chemokine C-C motif ligand; CXCL, chemokine C-X-C motif ligand; CCR, chemokine C-C motif receptor; CXCR, chemokine C-X-C motif receptor; rCCL17, recombinant chemokine C-C motif ligand 17; TREM, triggering receptor expressed on myeloid cells; STAT, signal transducer and activator of transcription; PPAR-γ, peroxisome proliferator-activated receptor-γ; Arg1, arginase 1; Ym-1, chitinase 3-like 3; FIZZ1, resistin-like-α; Nrf2, nuclear factor-erythroid 2 p45-related factor 2; VEGF, vascular endothelial growth factor; PDGF, platelet-derived growth factor; BDNF, brain-derived neurotrophic factor; Mt, functional mitochondria; HN, bioactive peptide humanin; AQP4, aquaporin-4; Cx43, connexin 43; PAR1, G protein-coupled receptor 1; Sox10, SRY-Box transcription factor 10; Th, T helper; RORγt, retinoic acid receptor-related orphan receptor γt; Treg, T regulatory; Foxp3, forkhead box P3; NETs, neutrophil extracellular traps.

### 2.1. Microglia/macrophages

Microglia, most of which evolved from erythroid precursor cells in the yolk sac (Borst et al., 2021), have long been thought to be responsible for the removal of debris generated during periods of neurological development and disease as the resident macrophages of the central nervous system (Prinz et al., 2019).

After cerebral hemorrhage, resting microglia are activated, and at the same time, macrophages derived from monocytes migrate from the blood to the CNS (Bai et al., 2020). Currently, the surface markers common to activated microglia/macrophages can be analyzed using Western blotting, enzyme-linked immunosorbent assay, real-time quantitative PCR, flow cytometry, and other experimental means, such as CD86, CD16 (Lan et al., 2017), and it has also been detected that such as CD206, are present only in macrophages (Miyake et al., 2022). Howbeit, since the two are indistinguishable in morphology and most functions, we will put them together in this review (Masato et al., 2017).

Microglia/macrophages are chiefly engaged in the process of neuroinflammatory cascade response to secondary injury in cerebral hemorrhage (Xue and Yong, 2020). Activated microglia/macrophages are typically classified into M1 (pro-inflammatory or classically activated) and M2 (anti-inflammatory or alternatively activated), M1 and M2 types are not fixed and can dynamically undergo phenotypic switching, but the exact mechanism is not clear (Xiong et al., 2016). The animal model of ICH shows that M1 type is beginning to rise within 3 h after the beginning of cerebral hemorrhage, peaks after 3 days, and returns to normal range slowly after a week (Zhang et al., 2016). In contrast, the M2 type is barely detectable in the ultra-early stages of cerebral hemorrhage and starts to rise after the third day, gradually reaching a peak after the seventh day, after which it slowly returns to normal values (Bi et al., 2021). The M2 phenotype can be divided into three subtypes-M2a, M2b, and M2c-which all have distinct cell surface markers and functions (Ohnishi et al., 2020). M2a primarily undertook inflammation inhibition and cell regeneration, M2b is both pro-inflammatory and anti-inflammatory, and M2c plays a major role in tissue remodeling and matrix deposition (Vibol Chhor et al., 2013; Lan et al., 2017; Tschoe et al., 2020).

#### 2.1.1. M1 (pro-inflammatory type)

Microglia are the most abundant innate immune cells in the CNS (Liu et al., 2021), Resting microglia are substantially entailed in angiogenesis and the development of the blood-brain barrier (da Fonseca et al., 2014). Early after the commencement of cerebral hemorrhage, the surface receptors CD16, CD32, CD68, CD86, and major histocompatibility complex II (MHCII) were differentially elevated in microglia (Hu et al., 2012; Xia et al., 2015; Tschoe et al., 2020; Bi et al., 2021) and can be used as markers to identify M1. Microglia/macrophages are activated into M1 type and secrete pro-inflammatory cytokines such as IL-1β, TNF-α, and reactive oxygen species (ROS) (Eva and Roland, 2014; Klebe et al., 2015; Tuttolomondo et al., 2020). IL-1β and TNF- α could activated Nucleotide-binding oligomerization domain 1 (NOD1)/receptor-interacting protein 2 (RIP2) signaling, which upholds microglia toward M1 and exacerbate inflammatory response (Wang M. et al., 2020). Macrophages stimulated by IFN-γ polarize into M1 type and produce reactive oxygen species (ROS) (Gordon and Taylor, 2005), which results in oxidative stress and aggravate secondary injury after ICH (Boche et al., 2013). Some studies have ascertained that during a secondary injury in cerebral hemorrhage, microglia/macrophages secrete a variety of interleukins concerned in the pro-inflammatory response, such as IL-1β, IL-2, IL-6, IL-12, and IL-23 (Hongwei et al., 2012; Vibol Chhor et al., 2013; Hu et al., 2014; Bai et al., 2020). They can activate endothelial cells and attract the aggregation of peripheral lymphocytes, neutrophils, and monocytes through chemotactic factors, which induce further expansion of inflammation (Mishra and Yong, 2016). Hongwei et al. (2012) has discovered that in the absence of an inhibitor of cytokine signaling 3 (SCOS3), macrophages are polarized toward the M1 phenotype, and gene levels of IL-6, IL-12, and IL-23 are elevated compared to normal levels (Qin et al., 2012), while IL-6-induced signal transduction and phosphorylation of transcriptional activator proteins STAT1 and STAT3 are enhanced, contributing to increased inflammation (Hongwei et al., 2012). This is due to the presence of SCOS3, which can bind to the Tyr759 region of gp130 protein (Sommer et al., 2005) and inhibit IL-6 signaling in macrophages (Kang et al., 2019).

Chemokines are a family of small-molecule peptides, divided into four paramount classes: C, CC, CX3C, and CXC, which have a recruiting effect on immune cells applied to the inflammatory response (Bajetto et al., 2001; Mirabelli-Badenier et al., 2011). Activated microglia/macrophages secrete cytokines such as lipopolysaccharide (LPS) and IL-1β, which in turn nourish their secretion of some pro-inflammatory chemokines, such as CCL3, CCL4, CXCL1 (Zhong et al., 2016; Bai et al., 2020). CXCL4 is one of the most consequential chemokines in the CXC subfamily, and there is evidence that the heterodimer CXCL4-CCL5 has appertained to the process of brain injury, and the addition of inhibitors can significantly enhance neurological function in mice (Vogel et al., 2014; Tuttolomondo et al., 2020). In addition, it has also been spotted that CCL20, CXCL2, and CXCL10 can accelerate the migration of peripheral immune cells (Shiratori et al., 2010; Guedj et al., 2014; Chong et al., 2015). In an autologous blood model of intracerebral hemorrhage in mice, elevated levels of CCL12 in the blood can result in aggregation of macrophages and T cells thereby exacerbating brain injury (Huang et al., 2020). In contrast, the chemokine receptors CCR1, CCR5, and CXCR4 present on the surface of microglia/macrophages can regulate their activation (Yu S. J. et al., 2020; Yan et al., 2021). For example, CCR1 expressed by microglia after brain hemorrhage can be activated through the CCR1/tetratricopeptide repeat 1 (TPR1)/extracellular signal-regulated kinase 1/2 (ERK1/2) signaling pathway, thereby promoting the development of neuroinflammation (Yan et al., 2020).

Matrix metalloproteinases are earthshaking components of the zinc-dependent metalloproteinase family and are pertained to the remodeling of the extracellular matrix (Yong et al., 2001). There is increasing evidence that matrix metalloproteinases are also associated with angiogenesis, inflammation development, and signaling pathways (Christopher and Kleifeld, 2006). After cerebral hemorrhage, M1 microglia/macrophages secrete swiftly MMP3, MMP9, and MMP12, which nurture inflammatory cell infiltration, engender neuronal apoptosis, and blood-brain barrier disruption (Power et al., 2003; Xue et al., 2009; Lattanzi et al., 2020).

Much attention has been paid to the role of Toll-like receptors (TLR) 2 and TLR4 in brain hemorrhage, a class of transmembrane signaling proteins that are primarily subsumed in non-specific immunity *in vivo* (Huang et al., 2013; Yan-Chun et al., 2014). In an autologous blood model in rats, Toll-like receptor 4-mediated nuclear factor-kappa B (NF-kB) signaling plays a key role in the process of inflammatory brain injury (Weiyu et al., 2009). The heme in erythrocytes was ferreted to enhance microglia activation *via* TLR4, thereby inducing NF-kB activation *via* the myeloid differentiation factor 88 (MyD88)/TIR-domain-containing adapter-inducing interferon-β (TRIF) pathway and causing an inflammatory response (Lin et al., 2012). MMP9 in astrocytes can be activated by TLR2 to damage the blood-brain barrier, enhance inflammatory cell infiltration, and induce inflammatory injury after cerebral hemorrhage (Min et al., 2015). TLR2/4 signaling is associated with a poor prognosis of cerebral hemorrhage and may provide effective targets for clinical treatment (Wang et al., 2013).

Activated microglia express triggering receptors expressed on myeloid cells 1 (TREM-1) on their surface, which belongs to the immunoglobulin superfamily. The main role of TREM-1 is to amplify the inflammatory response (Colonna and Facchetti, 2003). In an animal model of subarachnoid hemorrhage, TREM-1 can further deepen the neuroinflammatory response through NLRP3 inflammasome-mediated pyroptosis (Xu et al., 2021). Furthermore, Lu et al. (2021) has spotted that TREM-1 can bring about the polarization of microglia toward a pro-inflammatory phenotype and aggravate the inflammatory response through the PKC δ/CARD9 signaling pathway and by enhancing the activation of high mobility group protein B1 (HMGB1) on itself.

#### 2.1.2. M2 (anti-inflammatory type)

**FIGURE 1:**
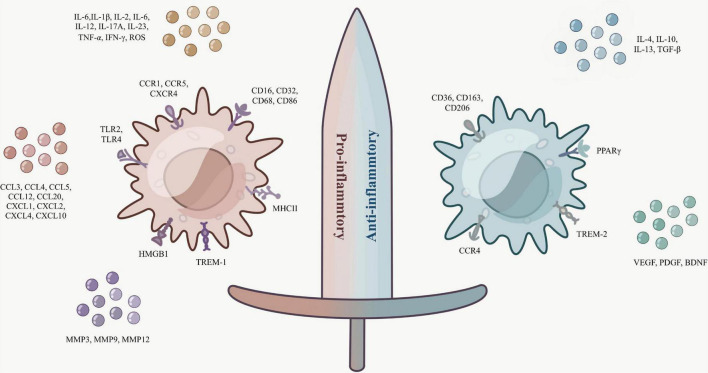
Microglia/macrophages are a double-edged sword. The surface markers and secretory factors of M1 (pro-inflammatory type) and M2 (anti-inflammatory type).

Microglia/macrophages act as a double-edged sword (Figure 1), polarizing toward suppression of inflammation in the middle and late stages of cerebral hemorrhage (Liu et al., 2021). Distinct from M1 surface markers, M2 microglia/macrophage surface receptors are mostly associated with inflammation inhibition and hematoma clearance, such as scavenger receptors CD36, CD47, CD91, phagocytosis receptor CD163, and mannose receptors CD206 (Jens et al., 2002; Wang et al., 2017; Jing et al., 2019).

As mentioned previously, M2 microglia/macrophages can be divided into M2a, M2b, and M2c subtypes based on surface receptors and function. The surface receptors of M2a are mostly CD36 and CD206, while there is a close association between the anti-inflammatory factors IL-4, IL-13, transforming growth factor β (TGF-β), and M2a (Yang et al., 2016). *In vitro* and *in vivo* studies have shown that IL-4 can encourage the phagocytosis of microglia/macrophages by activating STAT6 and IL-1 receptor-like 1 signaling (ST2) pathway, and enhance the phagocytosis of hematoma of M2a microglia/macrophages, and IL-4 may have a promising role in hematoma clearance in cerebral hemorrhage (Xu et al., 2020). The signaling pathway interferon-regulatory factor (IRF)/STAT is a momentous pathway for macrophages to polarize toward M1 or M2, and IL-4/13 activates this signaling pathway to polarize macrophages toward M2, thus exerting an inhibitory effect on inflammation (Sica and Mantovani, 2012; Wang et al., 2014). Little research is known about M2b, which is thought to have both pro-inflammatory and anti-inflammatory functions. It was determined that IL-10, CD86, and MHCII are substantially expressed by M2b microglia and have some effect on reducing the inflammatory response (Zhao H. et al., 2015). Diverse from the two previous ones, M2c is normally connected with synaptic remodeling and matrix deposition. IL-10 also plays a role in stimulating M2c, and the classical M2-type markers chitinase 3-like 3 (Ym-1), arginine 1 (Arg-1), and resistin-like-α (FIZZ1) are commonly rummaged in M2a and M2c, while the phagocytic receptor CD163 is more naturally seen in M2c (Mantovani et al., 2004; Boche et al., 2013; Feuerer et al., 2021; Sapudom et al., 2021).

Additionally, researchers have spotted that several elements with hematoma clearance, tissue repair, and inflammation inhibition are closely associated with M2-type microglia/macrophages. It was shown that LPS stimulates elevated microglia expression of peroxisome proliferator-activated receptor γ (PPARγ), a widely available transcription factor receptor-associated with inflammatory response and trophic factor production (Cai et al., 2018). The presence of PPARγ and nuclear factor-erythroid 2 p45-related factor 2 (Nrf2) presence can co-regulate CD36 expression and enhance phagocytosis of microglia/macrophages (Zhao X. et al., 2015). Distinguishable from TREM-1, TREM-2 expressed on the surface of activated microglia can attenuate neuroinflammation and amend neurological function *via* the phosphatidylinositol 3-kinase (PI3K)/protein kinase B (Akt) signaling pathway (Chen et al., 2020). Recombinant CCL17 (rCCL17)-dependent CCR4 can also achieve ameliorated neurological function after cerebral hemorrhage in part through PI3K/Akt/forkhead transcription factor 1 (Foxo1) signaling (Deng et al., 2021). A class of growth factors that tissue and inflammatory recoveries such as vascular endothelial growth factor (VEGF), platelet-derived growth factor (PDGF), and brain-derived neurotrophic factor (BDNF) are also receiving increasing attention for their role in M2 microglia/macrophages (Hu et al., 2014; Kanazawa et al., 2017; Liu et al., 2021).

### 2.2. Astrocytes

Astrocytes are the most numerous classes of neuroglia, which are predominantly interrelated with synapse formation and the establishment of the blood-brain barrier (Sofroniew, 2020). As a relatively complex functional immune cell in the nervous system, little is known about astrocytes. In the presence of pathological reactions such as injury and inflammation in the body, astrocytes are activated to produce neurotoxins, induce neuronal death, and advance the development of inflammation, and this type of astrocytes is called the A1 type. In contrast, type A2 astrocytes, which are traditionally induced to arise under ischemic conditions, can upregulate neurotrophic factor levels, incubate tissue repair, and have a protective effect (Liddelow et al., 2017; Mithilesh et al., 2018).

In this review, we focus on the role of astrocytes in intracerebral hemorrhage. CD147 receptor expressed on the surface of astrocytes is elevated after cerebral hemorrhage, and the suppression of CD147 can reduce the level of MMP-9 in blood and upgrade the prognosis of neurological function (Anthony et al., 2020). Aquaporin-4 (AQP4), a water channel protein of astrocytes, has a decreased expression after cerebral hemorrhage and is a determinant of perihematomal edema. Enhancing AQP4 expression may provide an effective therapeutic measure for the prevention of edema after cerebral hemorrhage (Zhang et al., 2019; Jeon et al., 2021). Jung et al. (2020) dug out that functional mitochondria (Mt) secreted by astrocytes can be taken up by microglia and induce the expression of bioactive peptide humanin (HN), which supports microglia/macrophage to polarize toward M2 and accelerate hematoma clearance. There is evidence that G protein-coupled receptor 1 (PAR1), located on the peduncle of astrocytes, is associated with the process of brain injury after cerebral hemorrhage (Rohatgi et al., 2004). What’s more, astrocytes secrete pro-inflammatory components such as IL-1β, IL-15, and TNFα, which aggravate the inflammatory response. On the other hand, they also secrete the anti-inflammatory members IL-6 and TGF-β, which boost neurological recovery (Allaman et al., 2011; Scimemi, 2018). The presence of a gap junction protein, connexin (Cx43), on the astroglial cell surface and the excessive opening of Cx43 hemichannels engenders the release of inflammatory factors in the inflammatory response (Yu H. et al., 2020).

### 2.3. Oligodendrocytes

As one of the neuroglia, oligodendrocyte progenitor cells (OPCs), which originate from neuroepithelial progenitor cells (NPCs) in the forebrain, are the source of oligodendrocytes. The differentiation of OPCs into oligodendrocytes is usually divided into two steps. In the initial step, OPCs differentiate into immature pre-OLs (pre-OLs). In the secondary step, pre-OLs further mature into myelinating oligodendrocytes, also known as mature oligodendrocytes (mature-OLs) (van Tilborg et al., 2018). At different stages of differentiation, oligodendrocytes express a variety of markers. However, some factors such as Olig1/2, NKx2.2, and (SRY-Box transcription factor 10) Sox10 are persistently present throughout oligodendrocyte maturation (Kang and Yao, 2019). For example, Olig1/2, expressed in the pMN region of the spinal cord, is a basic-helix-loop-helix (bHLH) transcription factor that is usually expressed together with NKx2.2 and abets oligodendrocyte differentiation in the spinal cord (Zhou and Anderson, 2002).

Oligodendrocytes are the cells with the highest iron content in the central nervous system, and iron overload is one of the ingredients of secondary injury in cerebral hemorrhage. Activation of TLR4 on the surface of macrophages in some studies lifts the replacement of oligodendrocytes by precursor cells, enhancing iron clearance and reducing brain injury (Goldstein et al., 2017). The presence of hematoma and inflammation after ICH begets white matter damage (Fu et al., 2021). As an eventful component of the white matter of the brain, oligodendrocytes die in considerable numbers and OPCs infiltrate into the white matter and differentiate into OLs, leading to myelin regeneration and restoration of neurological function (Joseph et al., 2016). Tmem10, a kind of new type I transmembrane protein, was espied to be specifically expressed in oligodendrocytes and can be used as a marker for the evaluation of myelin lesions (Jiang et al., 2014). In conclusion, the primary function of oligodendrocytes in brain hemorrhage is to participate in the regeneration of myelinated axons after injury.

### 2.4. Lymphocytes

Lymphocytes are a class of cell lines with immune recognition functions and are routinely classified into T lymphocytes, B lymphocytes, and natural killer (NK) cells based on their functions (Peter et al., 2000). Currently, most studies on lymphocytes in brain hemorrhage are on T cells, and studies on B cells and NK cells in brain hemorrhage are relatively rare (Mracsko et al., 2014).

Helper T (Th) cells are the most common type of T cells pertaining to cerebral hemorrhage, and at least four subtypes of Th1, Th2, Th17, and Treg have been identified, each mediating a different immune response (Zhu and Paul, 2008). Characteristic markers of Th1 cells subsume the transcription factors T-bet, IFN-γ, and IL-2, which have to do with cellular immunity (Bretscher, 2019). Th2 secretes IL-4, IL-5, IL-9, and IL-13, among which IL-4 induces B lymphocytes to secrete immunoglobulin IgE and participate in humoral immune response, IL-4 and IL-13 prompt macrophages to polarize to M2 type and IL-5 results in an increase in eosinophils (Walker and McKenzie, 2018). Th1 and Th2 can mutually inhibit each other’s differentiation by secreting cytokines (Jinfang and William, 2010).

Th17 specifically secretes cytokines such as IL-17, IL-21, IL-22, IL-23, and TGF-β and the transcription factor retinoic-acid-receptor-related orphan receptor γt (RORγt) (Dong, 2008; Zúñiga et al., 2013), which are substantial for organismal immune regulation and immune-mediated diseases (Rajatava et al., 2013). For example, the pro-inflammatory factor IL-23 can bring about upregulation of IL-22 secreted by Th17 cells and regulate the effector function of Th17 cells (Stockinger and Veldhoen, 2007). In contrast, T regulatory cells can be specifically recognized based on the surface receptors CD25, CD127, and the transcription factor forkhead box p3 (Foxp3) (Peck and Mellins, 2010; Kleinewietfeld and Hafler, 2013). *In vitro* pilot study, Yang et al. (2014) detected that Treg cells might activate NE-kB *via* the C-Jun N-terminal kinase (JNK)/extracellular signal-regulated kinases (ERK) pathway, thereby inhibiting microglia-mediated inflammatory responses.

### 2.5. Neutrophils

Neutrophils recruited from peripheral blood to the area of cerebral hemorrhage could secrete TNF-a, ROS, and MMP-9, which further intensify brain injury (Maestrini et al., 2020; Xue and Yong, 2020). Ranran et al. (2021) scouted up an association between neutrophil secretion of neutrophil extracellular traps (NETs) and tissue plasminogen activator (tPA) thrombolysis-induced cerebral hemorrhage. NETs mediate the disruption of the blood-brain barrier by IFN-β and IL-6 through the cyclic GMP-AMP synthase (cGAS)-STING pathway, exacerbating tPA-induced cerebral hemorrhage (Ranran et al., 2021).

## 3. Crosstalk between different immune cells

**FIGURE 2:**
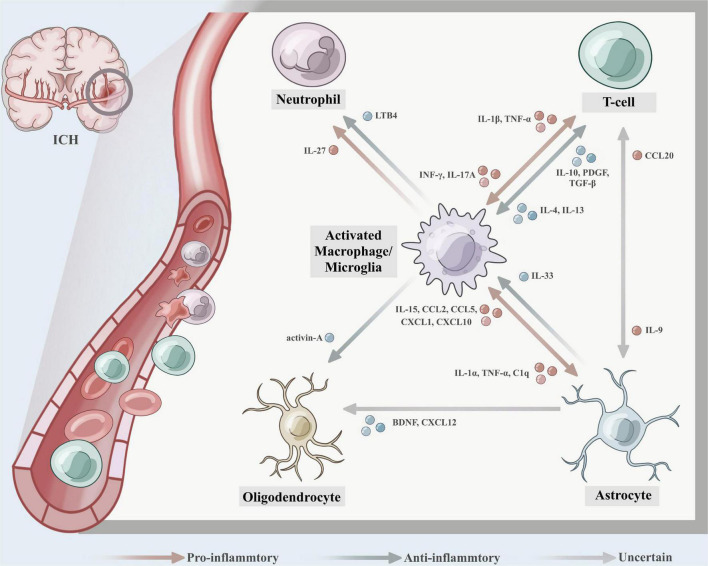
The crosstalk between different immune cells after intracerebral hemorrhage (ICH). (1) Activated microglia were able to induce astrocyte conversion to A1 type by secreting IL-1α, TNF, and C1q, promoting neuronal death and exacerbating the inflammatory injury, astrocytes express IL-15, CCL2, CCL5, CXCL1, and CXCL10, which enabled microglia to polarize toward pro-inflammatory phenotype. While astroglial-derived IL-33 promotes microglia synaptic phagocytosis and neural circuit development, which maintains synaptic homeostasis in the developing central nervous system. (2) Th1 cells secrete IFN-γ to promote macrophage to polarize toward M1, and the activated macrophages further secrete inflammatory factors IL-1β and TNF-α, which cause aggregation of Th17 cells, exacerbating the inflammatory response and leading to tissue damage. IL-17A expressed on Th17 cells was detected to induce microglial activation and inflammatory responses in a mouse autologous blood model, while mediating autophagy of microglia. Th2-driven IL-4 and IL-13 promote macrophages to M2-type polarization, which secret IL-10, PDGF, and TGF-β contributing to collagen deposition and tissue healing. (3) M2 microglia/macrophage-derived activin-A boosts oligodendrocyte differentiation, and astrocyte-derived brain-derived neurotrophic factor (BDNF) contributes to the differentiation of oligodendrocyte progenitor cells (OPCs) into oligodendrocytes and may be an effective therapeutic target for the repair of white matter damage after ICH. (4) Leukotriene B4 (LTB4) derived from microglia causes neutrophils to migrate to the hematoma site and exacerbates the inflammatory damage effect, while microglia-derived IL-27 can mediate the functional regulation of neutrophils, inhibit the production of inflammatory factors by neutrophils, and reduce the formation of hematoma and edema. (5) IL-9 produced by Th cell subsets could exacerbate disease progression by inducing astrocytes to secrete CCL20.

A growing number of studies have nosed out that changes in the inflammatory response after brain hemorrhage are not only the result of an individual immune cell but also associated with interactions between different immune cells (Figure 2). Crosstalk between microglia and astrocytes is a hot spot and frontier in immune cell research, and their mutual influence on each other’s phenotype and function are worthy of attention in the field of neurological diseases, especially in ICH (Mithilesh et al., 2018; Liu et al., 2020). On the one hand, it has been shown that activated microglia were able to induce astrocyte conversion to A1 type by secreting IL-1α, TNF, and C1q, promoting neuronal death and exacerbating inflammatory injury (Liddelow et al., 2017). Shi et al. (2020) studied the relationship between astrocytes and microglia using transgenic C57BL/6 mice targeting IL-15 expression in astrocytes (GFAP-IL-15tg) and showed that astrocytes targeting IL-15 expression enabled microglia to polarize toward pro-inflammatory phenotype. Brain edema and neurological functional scores in GFAP-IL-15tg brain hemorrhage mice were significantly boosted after using the microglia-depleting agent PLX3397 (Shi et al., 2020). On the other hand, Vainchtein et al. (2018) detected that astroglial-derived IL-33 assists microglia synaptic phagocytosis and neural circuit development, which maintains synaptic homeostasis in the developing central nervous system. The crosstalk between microglia and astrocytes to a certain extent facilitates the proliferation of microglia, transition to M2, and the polarization of astrocytes to A2, which play a neuroprotective role (Kim and Son, 2021). In addition to secreting cytokines, astrocytes can also express a large array of chemokines by recognizing different microorganisms, such as CCL2, CCL5, CXCL1, CXCL10 (McKimmie and Graham, 2010). For example, astrocyte-secreted CCL2 was able to uphold microglia migration and polarization toward the pro-inflammatory phenotype *via* the CCL2/CCR2 pathway, and the use of RS102895, an inhibitor of CCR2, significantly fortified neuroinflammatory symptoms (He et al., 2016).

The crosstalk between other immune cells and microglia is also gradually gaining attention from researchers. Miron et al. (2013) identified that M2 microglia/macrophage-derived activin-A boosts oligodendrocyte differentiation, invokes myelin regeneration, and could be a potential target for the treatment of white matter injury after cerebral hemorrhage. Microglia/macrophages interact with Th cells to stake phenotypic switching (Klebe et al., 2015). Th1 cells secrete IFN-γ to cultivate macrophages to polarize toward M1. In the meantime, the activated macrophages further secrete inflammatory factors IL-1β and TNF-α, which cultivate aggregation of Th17 cells and neutrophils, exacerbating the inflammatory response and leading to tissue damage. Likewise, IL-17A was detected to induce microglial activation and inflammatory responses in a mouse autologous blood model, while mediating autophagy of microglia (Shi et al., 2018). Unlike Th1, Th2-driven IL-4 and IL-13 champion macrophages to M2-type polarization, which secret IL-10, PDGF, and TGF-β contributing to collagen deposition and tissue healing (Gordon, 2003; Prasse et al., 2007; Murray and Wynn, 2011). As previously mentioned, Treg cells inhibit the secretion of inflammatory factors TNF-α, IL-1β, and MMP-2 by microglia through the JNK/ERK pathway and the activation of NF-κB, and attenuate the inflammatory response after intracerebral hemorrhage (Yang et al., 2014). Hijioka et al. (2020) rummaged that leukotriene B4 (LTB4) is mainly derived from microglia. The LB4-BLT signaling axis forwards the activation of inflammatory microglia, breeds neutrophils to migrate to the hematoma site, and worsens the inflammatory damage effect (Hijioka et al., 2017). While Zhao et al. (2017) showed that microglia-derived IL-27 can mediate the functional regulation of neutrophils, inhibit the production of inflammatory factors by neutrophils, and reduce the formation of hematoma and edema.

To some extent, the crosstalk among astrocytes, oligodendrocytes, and Th cells is also present. *In vitro* and *in vivo* studies have shown that astrocyte-derived BDNF engenders the differentiation of OPCs into oligodendrocytes and may be an effective therapeutic target for the repair of white matter damage after ICH (Miyamoto et al., 2015). CXCL12, a chemokine expressed by astrocytes, is also associated with oligodendrocytes. Patel et al. (2012) ferreted that the signal tumor necrosis factor receptor 2 (TNFR2) in astrocytes in a demyelinating mouse model was able to induce auto-secretion of CXCL12, causing proliferation and differentiation of OPCs and myelin regeneration. In experimental autoimmune encephalomyelitis (EAE) disease, Zhou et al. (2011) ascertained that IL-9 produced by Th cell subsets could magnify disease progression by inducing astrocytes to secrete CCL20, thereby causing Th17 cells to recruit to brain tissue. Whether these associations occur in cerebral hemorrhage deserves to be explored in depth.

## 4. Immunotherapies for intracerebral hemorrhage

At the end of this review, we concluded the clinical use of selected immunotherapeutic agents targeting neuroinflammation after ICH, with the expectation that they might provide useful clues for translational research on intracerebral hemorrhage from the basic to clinical. Please refer to Table 2.

**TABLE 2:** Current and potential clinical medicine for intracerebral hemorrhage (ICH).

Drug	ClinicalTrials.gov identifier	Trial acronym	Target	Development phase	Status
Minocycline	NCT01805895	MACH	Various	Phase 1/2	Completed
Fingolimod	NCT04088630	FITCH	S1PR	Early phase 1	Ongoing
Siponimod (BAF312)	NCT03338998	/	S1PR	Phase 2	Terminated
Stains	NCT04857632	STATIC	Various	Phase 2/3	Ongoing
Stains	NCT03936361	SATURN	Various	Phase 3	Ongoing
Deferoxamine	NCT02175225	iDEF Ttrial	Iron	Phase 2	Completed
IL-1Ra	NCT03737344	BLOC-ICH	IL-1 receptor	Phase 2	Completed
CN-105	NCT03168581	CATCH	apoE	Phase 2	Completed

We searched www.ClinicalTrials.gov using the keyword “intracerebral hemorrhage” and found 316 clinical trial registrations as of 6 February 2022. From the 316 entries, drugs of relevance to immunotherapy were selected to form this table. abbreviationS1PR, sphingosine 1-phosphate receptor; IL-1Ra, IL-1 receptor antagonist; apoE, apolipoprotein E.

Minocycline is a wide spectrum of tetracycline-type antibiotics. Preclinical trials have shown that minocycline reduces the levels of MMP-9 and MMP-12 after cerebral hemorrhage (Power et al., 2003; Wasserman and Schlichter, 2007; Chang et al., 2017), assists microglia polarization from M1 to M2 type, and facilitates the reduction of inflammatory damage and demyelination of white matter after ICH (Moller et al., 2016; Yang et al., 2019). A single-blind randomized controlled study of minocycline (MACH) showed that minocycline was safe for humans and had a neuroprotective effect by reducing inflammatory markers. However, oral administration did not reinforce the patient’s 90-day functional prognosis. The authors considered that the mode of administration was not conducive to patient absorption and delayed the onset of drug action (Fouda et al., 2017; Malhotra et al., 2018).

Fingolimod (FTY720) and siponimod (BAF312) are sphingosine 1-phosphate receptor (S1PR) modulators (Bobinger et al., 2019; Wang Z. et al., 2020). S1PRs on the surface of microglia are linked with inflammatory regulation and accelerate microglia polarization toward M2, which has the effect of reducing brain tissue edema and neuroprotection (Zhang et al., 2021). A double-blind, placebo-controlled clinical trial (FITCH) of fingolimod is ongoing, while the first clinical trial of Siponimod (NCT03338998) has been terminated after interim data analysis due to no potential efficacy observed.

Statins can be used to treat intracerebral hemorrhage by reducing the expression of inflammatory markers and increasing PPAR-γ activity (Ewen et al., 2013; Wang et al., 2018). Clinical trials STATIC and SATURN on statins are currently underway. Other drugs for ICH such as Deferoxamine, IL-Ra, and CN-105 are gaining attention from researchers. Deferoxamine reduces oxidative stress, tissue damage after stroke, and elevates the function of M2 microglia (Xie et al., 2014; Farr and Xiong, 2021). IL-1 receptor antagonist (IL-Ra) suppresses the pro-inflammatory activity of IL-1, and CN-105 competes with apolipoprotein E (apoE) to regulate the immune response after cerebral hemorrhage (Tu et al., 2017). Related clinical trials are in progress.

## 5. Outlook

As the Internet has entered the era of the Internet of Everything, the research of neuroinflammation after brain hemorrhage is expanding from a single immune cell or molecule to multiple immune cells or molecules. Different immune cells interact with each other and are intertwined into a network that acts at different stages after intracerebral hemorrhage. Immunotherapeutic drugs for brain hemorrhage have also sprung up. In addition to focusing on new drugs, researchers are also working on innovations in the way drugs are delivered. Guo et al. (2019) invented the intranasal charge-driven delivery system for the charge-driven antihypertensive drug nicardipine to treat cerebral hemorrhage in rats and achieved significant outcomes. Dharmalingam et al. (2020) used a combination of iron chelator (Deferoxamine, DFO) and antioxidant hydrophilic carbon clusters (HCCs) to develop a multifunctional DFO-PEG-HCC nanoparticle, which demonstrated the ability to reduce ROS accumulation and prevent cellular aging after ICH in an animal model. We believe that as the research on the mechanism of ICH deepens, more and more potential therapeutic drugs will be developed, which will be of great benefit to patients with intracerebral hemorrhage. Figures were created with Biorender software, ©www.biorender.com.

## Author contributions

WZ and QW wrote this manuscript together. SH revised and provided advice on the writing of this manuscript. SC provided the general framework, wrote points about this review, and guided the accomplishment of the manuscript. All authors have contributed to this article and agreed to submit this version.
